# Supplementary material for: Statistical simulations show that scientists need not increase overall sample size by default when including both sexes in in vivo studies
Source: PLoS Biol. 2023 Jun 8;21(6):e3002129. doi: 10.1371/journal.pbio.3002129 (PMC10284409; doi:10.1371/journal.pbio.3002129)
Supplement: S1 Case Study — This supplementary case study is intended as an example pipeline for analysing data from in vivo experiments collected from both sexes. It is not intended as an exhaustive tutorial, and there are other appropriate methods for analysing the type of data that we are presenting. (DOCX) [file pbio.3002129.s001.docx]

**S1 Case Study: Example application of factorial analysis for data collected from two sexes**

This supplementary case study is intended as an example pipeline for analysing data from *in vivo* experiments collected from both sexes. It is not intended as an exhaustive tutorial, and there are other appropriate methods for analysing the type of data that we are presenting. For more discussion and guidance please consult relevant textbooks.

**Biological background**

The murine nephrotoxic nephritis model (NTN) recapitulates multiple features of glomerulonephritis, a form of chronic kidney disease, and is induced via administration of antibodies targeted against the murine glomerular basement membrane (GBM) (referred to as nephrotoxic serum (NTS)) (1,2). This model may display a differential induction effect by sex, with female mice reported to display greater susceptibility to NTS (3). Therefore, we selected this model as a pilot to investigate an optimised analysis pipeline for analysis of data including two sexes. We subsequently performed an experimental study where animals of each sex were allocated using an experimenter-masked (blinded) minimisation randomisation procedure, based on body weight, to treatment groups. Notably in the clinic, anti-GBM disease (Goodpasture Syndrome) has historically been described as male-dominated, however, this has been disputed in more recent investigations, where no sex effect was shown (4).

*Step 1: visualisation*

As sex should be considered a primary factor of experimental interest, data should be visualised by sex separately for each treatment level. Best practice is to present individual datapoints from each animal, with indications of central tendency and variance for each treatment group (e.g., boxplot). Here we present the Urinary Albumin-to-Creatinine (UACR) (S1 Fig 1A) alongside gene expression data obtained from mice treated with either control (serum) or NTS, separated by sex (S1 Fig 1B).


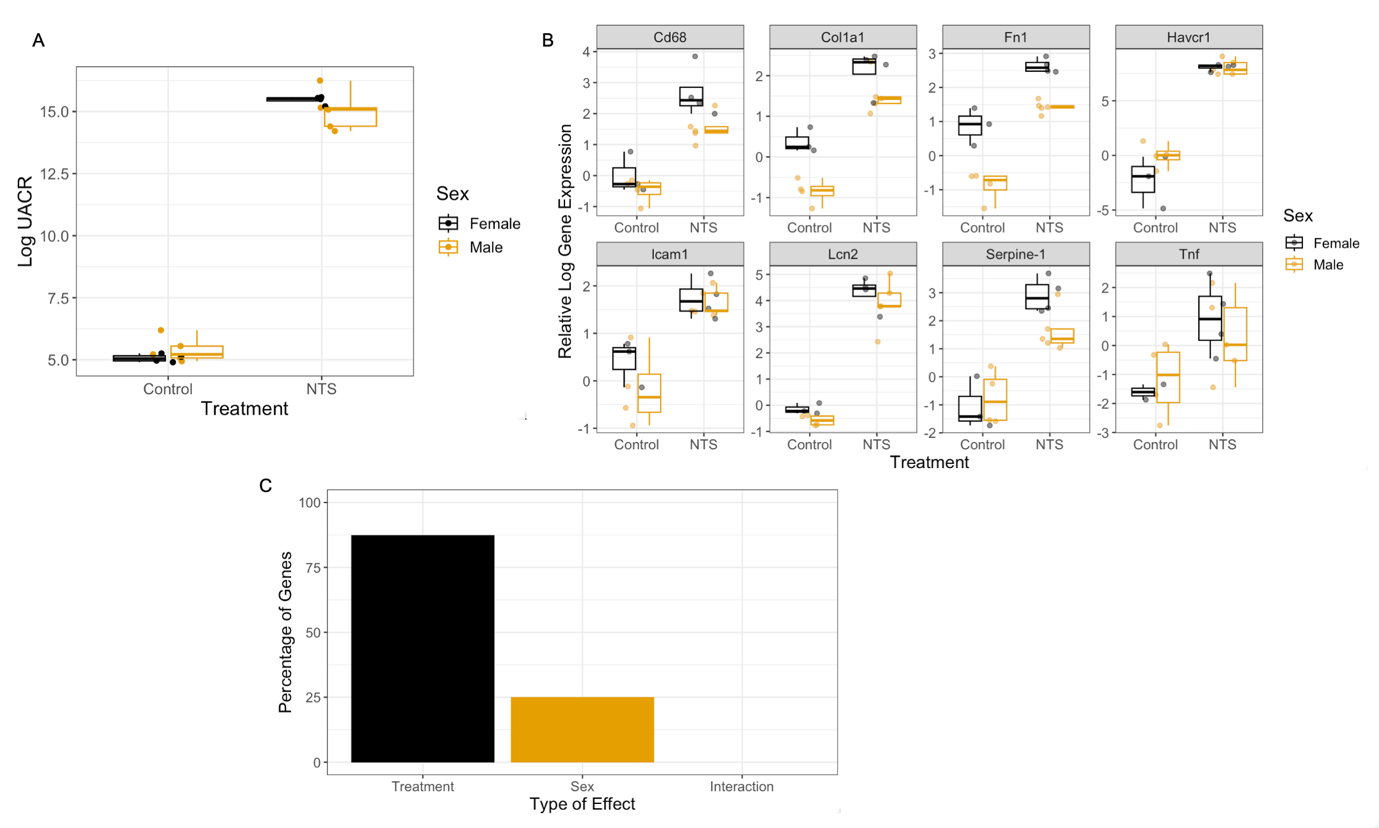


*S1 Figure 1: Visualisation of data by treatment and sex in the NTN model of kidney disease. A) Urinary Albumin-to-Creatinine Ratio (UACR) at day 4 post-dosing (N = 5 per treatment group). B) Relative expression levels for all measured genes (up to N = 5 per condition due to some missingness attributable to qPCR technical issues). C) Overall summary of statistically significant model effects (Benjamini-Hochberg corrected p*-value < 0.05*) in the dataset. The data underlying this Figure can be found in* [*https://doi.org/10.5281/zenodo.7806724*](https://doi.org/10.5281/zenodo.7806724)*.*

*Step 2: two-way ANOVA*

Two-way ANOVA is a regression analysis technique that handles factorial experimental designs, where the scientific hypothesis is a change in means between groups, by partitioning the variance that is attributable to each factor in the data independently prior to estimating the interaction effect.

This case study is a factorial design, as there are two factors of interest: the treatment and sex. In this example, the treatment has two levels (NTS and control, and sex has two levels (male and female). Note, this analysis method is not restricted to two levels for a factor; the treatment could have been a treatment with four levels (vehicle, low, medium, and high dose). However, it is restricted to examples where the residuals (variance components “left over” following partitioning the variability attributable to main effects and interactions) are approximately normally distributed and linear and that the measures are independent and continuous.

In the example dataset, for UACR, there was a significant effect of NTS treatment (F(1, 14) = 1685.979, *p* < 0.0001). There was no significant main effect of sex (F(1, 14) = 0.045, *p =* 0.835) or treatment by sex interaction (F(1, 14) = 2.451, *p* = 0.14) (S1 Fig 1C). Therefore, we did not detect any significant sex-related effects on the primary measure of model induction.

| **Gene: *Col1a1*** | Degrees of freedom | Sum of squares | Mean squares | F-value | P-value |
| --- | --- | --- | --- | --- | --- |
| Treatment | 1 | 17.629 | 17.629 | 93.961 | 0.0000005 |
| Sex | 1 | 2.986 | 2.986 | 15.917 | 0.00179 |
| Treatment * Sex | 1 | 0.409 | 0.409 | 2.178 | 0.16577 |
| Residuals | 12 | 2.251 | 0.188 |  |  |
| **Gene: *Lcn2*** | Degrees of freedom | Sum of squares | Mean squares | F-value | P-value |
| Treatment | 1 | 77.88 | 77.88 | 185.195 | 0.000000011 |
| Sex | 1 | 0.72 | 0.72 | 1.723 | 0.214 |
| Treatment * Sex | 1 | 0.00 | 0.00 | 0.00 | 0.995 |
| Residuals | 12 | 5.05 | 0.42 |  |  |

*S1 Table 1: Example two-way ANOVA output when testing the three model terms (treatment, sex and interaction). Shown are genes Col1a1, which exhibited both a significant main effects of treatment and sex, and Lcn2, which exhibited a significant main treatment effect following NTS administration. Neither gene exhibited a significant treatment by sex interaction.*

For the panel of genes, each gene was analysed independently by two-way ANOVA. An example 2-way ANOVA output is displayed in S1 Table 1. These results highlight that both genes exhibit a significant main effect of treatment. For *Col1a1*, there is also a significant main effect of sex.

Alongside statistical significance, it is important to evaluate effect sizes to interpret the results in their biological context. A common method for this is to examine contrasts through changes in estimated marginal means. An example output from this method of analysis is provided in S1 Table 2.

| **Gene: *Col1a1*** | **Treatment** | Estimated marginal mean | SE | df | Lower CL | Upper CL |
| --- | --- | --- | --- | --- | --- | --- |
|  | Control | -0.238 | 0.165 | 12 | -0.598 | 0.123 |
|  | NTS | 1.822 | 0.145 | 12 | 1.506 | 2.139 |
|  | **Contrast** | Estimated change | SE | df | T ratio | P-value |
|  | Control-NTS | -2.06 | 0.22 | 12 | -9.358 | <0.0001 |
| **Gene: *Lcn2*** | **Treatment** | Estimated marginal mean | SE | df | Lower CL | Upper CL |
|  | Control | -0.365 | 0.248 | 12 | -0.904 | 0.175 |
|  | NTS | 4.075 | 0.218 | 12 | 3.602 | 4.549 |
|  | **Contrast** | Estimated change | SE | df | T ratio | P-value |
|  | Control-NTS | -4.44 | 0.33 | 12 | -13.472 | <0.0001 |

*S1 Table 2: Example estimated marginal means analysis for treatment terms. Estimated means are presented alongside the estimated change, with standard errors and CL (confidence limits).*

The estimated changes suggest large, generalisable mean shifts for both genes following treatment (averaging across sexes). This is consistent with the metrics of significance observed from the ANOVA output, but the size of the changes, alongside estimates of variability, provide additional biological context which assists with interpretation.

Since multiple independent statistical tests are being conducted (one ANOVA for each of the nine genes in the panel), there is the potential for accumulation of false positives and hence adjustment for multiple testing needs to be applied. Multiple methods exist (5), with varying approaches to manage the risk. The method selected should be based on the research hypothesis in question (see <https://multipletesting.com/analysis> for a freely accessible tool). In this example, we have implemented the Benjamini-Hochberg procedure, as it balances robust control of the false discovery rate with adequate maintenance of statistical power (6). The statistically significant results are displayed in S1 Table 3.


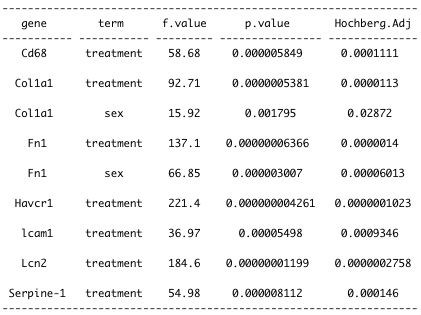


*S1 Table 3: Significant results from two-way ANOVA on the gene panel (N= 8 genes). For each gene, the degrees of freedom were DF(1, 12). The BH-adjusted p-values are presented alongside the original p-values.*

A summary of the significant model effects in the dataset is highlighted in S1 Table 3. Several genes exhibited a significant main effect of treatment. Notably, no statistically significant treatment by sex interaction effects were detected. A minority of genes demonstrated a significant baseline sex difference. We expect that this will be a typical result in many studies. The study was not powered to detect an interaction between treatment and sex, rather to give an indication of any large sex divergences in treatment effect, that may merit further investigation, were present. This is in line with the current SABV philosophy and guidance. The study has estimated the average treatment effect across the two sexes, thus is more generalisable than a study with only a single sex as we have explored a wider inference space.

Additionally, when using any statistical method, it is critical to check that the assumptions of the method are satisfied. In the case of ANOVA, independence of observations, normality and linearity of residuals and approximate equality of variances are the key assumptions. Though robust to some violation of assumptions, there is a risk of false inference if there are substantial deviations from the assumptions. To check the assumptions, the residuals may be plotted against the actual values to evaluate linearity and visualised on a Q-Q plot to assess for normality.

If the residuals do not conform to the linearity and normality assumptions, a typical strategy is attempting a transformation of the data to improve adherence. Frequently used transformations may include log and square root. In the case that the assumptions of ANOVA are violated, other modelling strategies (e.g., generalized linear modelling, non-linear models) may be more appropriate.

*Step 3: post-hoc tests*

The choice of whether to conduct post-hoc tests depends on the biology in question. For example, where a model of disease is being induced, it could be appropriate to estimate the treatment effect from the ANOVA alone, as a generalisable estimated effect. Conversely, it could be important to precisely understand the magnitude of the treatment effect in both sexes individually. For example, when there is a known difference in the biology between sexes, it would then be appropriate to estimate the effect in each sex independently using post-hoc tests.

In the presented case study, no treatment by sex interactions were detected. However, where a two-way ANOVA analysis yields a significant interaction, it is typical to additionally carry out pairwise post-hoc tests of significance to estimate the treatment effect within each sex and compare the size and direction of these effect to explore the biological difference that is responsible for the statistical interaction. A frequently committed error at this stage where two sexes are included is to misidentify the appropriate pairwise comparisons. For example, researchers often erroneously compare the means of treated males to treated females which conflates baseline sex differences with differences resulting from treatment. The risk from this error is high, as baseline sex differences are common (7). Another consequence of this error is the inflation of the false positive multiplicity burden by carrying out irrelevant comparisons (e.g., treated males to untreated females). Although there may be situations where a scientific hypothesis merits other comparisons we recommend the post-hoc tests compare untreated to treated groups within each sex where the interaction term is statistically significant and of biological interest. Importantly, where the number of treatment levels increases, post-hoc comparisons should also generally undergo adjustment for multiple testing, otherwise the risk of producing spurious false significance is greatly inflated.

***In vivo* experiments**

*Ethics Statement*

All animal experiments were conducted in accordance with the United Kingdom Animal (Scientific Procedures) Act 1986 and associated guidelines, approved by institutional ethical review committees (Alderley Park Animal Welfare and Ethical Review Board; Babraham Institute Animal Welfare and Ethical Review Board) and conducted under the authority of the Home Office Project Licences (PF344F0A0). All animal facilities have been approved by the United Kingdom Home Office Licensing Authority and meet all current regulations and standards of the United Kingdom.

*Animals, housing and husbandry*

*C57BL6N* mice (10 male and 10 female; age 6 weeks; Taconic France) were housed in groups of five mice per cage with an inverse 12 hours day-night cycle in a controlled temperature (19-23 °C) and humidity (45±65%) room as directed in the Code of Practice for the Housing and Care of Animals Bred, Supplied or Used for Scientific Purposes. Animals were housed in individually ventilated cages (IVC) and received food (Special Diet Services R&M No 1) and water ad libitum. Within the cage, the bedding material composed Aspen Eco-pure wood chips, nesting of sizzle nest and hemp happi-mats; as environmental enrichment cardboard smart home, cardboard or red polycarbonate tunnel, and Aspen chew stick. Animal health was checked twice daily from the day of delivery and water changed twice weekly.

*Procedures*

All procedures were carried out in a laminar flow hood. Male mice were always handled first when doing any procedures and hands cleaned with ethanol after handling each cage group to remove any traces of scent/odour from the previous cage to avoid any male aggression/fighting occurring. This approach introduces the risk of order effects but was implemented due to the welfare needs. The risk of order effects is minimised by maintaining the order during sample collection and processing.

Chip implantation: After a one-week acclimatization period, an identification chip was implanted by subcutaneous injection to identify the individual animals.

Urine collection: Urine was collected on day 4. For this, mice were placed in empty cages for a period of maximal 30 min. Each mouse had assigned one cage for urine collection which was used for the whole duration of the study. After collection, urine was transferred to collection plates and stored at –80 °C until analysed.

Nephrotoxic nephritis: Five male and five female mice were either injected with vehicle (control serum; Probetex; PTX) or nephrotoxic serum (Probetex; PTX001S-MS). For this, sera were diluted 1:1 with phosphate-buffered saline and injected intravenously into the tail vein on two consecutive days (day 0 and day 1) at a dose of 5ml/kg bodyweight.

Animals were randomly allocated to a treatment group by use of an in-house minimisation randomisation tool (8) to ensure balanced distribution of body weight across the treatment groups. During the randomisation step, the staff were masked (blinded) to the treatment information. During intervention, the support staff were aware of the treatment they were applying. During the conduct of the experiment the cages were not labelled with the treatment information and a common welfare management plan was implemented for all treatment groups. During the outcome assessment the samples were labelled with an identification number masking both the treatment and group information. During the analysis data was labelled with treatment group. All data was included in analysis. The analysis followed the analysis plan predefined in the experiment planning stage. At the end of the study, mice were euthanised by cervical dislocation followed by cardiac exsanguination prior to the collection of tissues.

Sample size justification:

In-house historic data was used to estimate the expected variance for a range of experimental parameters of interest. The final sample size was selected as it balanced the ability to detect treatment effects with practical considerations across the range of parameters. Specifically, we estimated power in terms of standard deviation unit change using the two-sample *t*-Test calculator of the Russ Lenth power tool (9). In this calculation, we estimated power by approximating the main effect as a two-tailed two group comparison at the 0.05 significance threshold and found that a N of 10 per treatment group (5 males and 5 females) would give us 0.8145 power to detect a change equivalent to 1.35 standard deviation units.

***Sample analyses***

*UACR analysis*

To determine the UACR, urine was thawed on ice and subsequently, centrifuged 3 min at 4000 x g at 4 °C. Urinary albumin was quantified by using a mouse albumin ELISA kit (ALPCO) according to the manufacturer's instructions. Urinary creatinine was measured by using a creatinine assay kit (Abcam) according to the manufacturer's instructions. Albumin was normalized to creatine levels and represented as mg/g.

*RNA isolation, cDNA synthèses and qRT-PCR analyses*

Total RNA was extracted with a MagMAX-96 Total RNA Isolation Kit (Cat #AM1830) and a KingsFisher 96 Flex system (Thermo Fisher Scientific) according to the manufacturer's instructions. For this, snap-frozen kidneys were homogenized and lysed in MagMAX-96 lysis buffer using a TissueLyser II machine (Qiagen). RNA quality and concentration were determined by a NanoDrop-1000 spectrophotometer (Thermo Fisher Scientific). One microgram of total renal RNA was transcribed to cDNA using the High-Capacity RNA-to-cDNA kit (Applied Biosystems). Quantitative analysis of target mRNA expression determined by TaqMan method using the Fast Advanced Master Mix (TaqMan) and was calculated using the double delta cycle threshold values (ΔΔCT) method. TaqMan duplex analyses were conducted using an QuantStudio 12K Flex Real-Time PCR system (Life Technologies). Gene expression levels were normalized to glyceraldehyde-3-phosphate dehydrogenase (*Gapdh*). TaqMan probe IDs are provided below in S1 Table 4.

| ***Gene*** | **TaqMan probe IDs** | **Dyes** |
| --- | --- | --- |
| *Gapdh* | Mm99999915_g1 | VIC |
| *Cd68* | Mm03047343_m1 | FAM |
| *Col1a1* | Mm00801666_g1 | FAM |
| *Fn1* | Mm01256744_m1 | FAM |
| *Havcr1* | Mm00506686_m1 | FAM |
| *Icam1* | Mm00516023_m1 | FAM |
| *Lcn2* | Mm01324470_m1 | FAM |
| *Serpine-1* | Mm00435858_m1 | FAM |
| *Tnf* | Mm00443258_m1 | FAM |

S1 Table 4 : TaqMan probes

**Statistical analysis of *in vivo* data**

The observed UACR data measured on day 4 post-dosing were log2 transformed prior to analysis by two-way ANOVA. Gene expression data obtained by qPCR were normalised to a housekeeping gene (*Gapdh*) to derive the double delta cycle threshold values (ΔΔCT). Subsequently, data were log2 transformed, and statistical analysis was applied. Data were analysed by two-way two-tailed ANOVA using type-II sum of squares, with treatment and sex as between-subject factors. Cage was not included as a factor in the model. This position was taken after minimizing practical cage differences (e.g., cage positions in rack) and the random allocation of animals to cage. All *p*-values derived from the analysis of the qPCR data were pooled and then corrected for multiple comparison by the Benjamini-Hochberg method (6) using the R package “multtest”. All analysis was carried out using R version 4.1.1 in Rstudio ([www.r-project.org](http://www.r-project.org)).

**References**

1. Nagai H, Yamada H, Nishigaki T, Nakazawa M, Koda A. The susceptibility of experimental glomerulonephritis in six different strains of mice. J Pharmacobiodyn. 1985 Jul;8(7):586–9.

2. Xie C, Sharma R, Wang H, Zhou XJ, Mohan C. Strain distribution pattern of susceptibility to immune-mediated nephritis. J Immunol. 2004 Apr 15;172(8):5047–55.

3. Ougaard MKE, Kvist PH, Jensen HE, Hess C, Rune I, Søndergaard H. Murine Nephrotoxic Nephritis as a Model of Chronic Kidney Disease. Int J Nephrol. 2018;2018:8424502.

4. Beckwith H, Lightstone L, McAdoo S. Sex and Gender in Glomerular Disease. Seminars in Nephrology. 2022 Mar 1;42(2):185–96.

5. Chen SY, Feng Z, Yi X. A general introduction to adjustment for multiple comparisons. J Thorac Dis. 2017 Jun;9(6):1725–9.

6. Benjamini Y, Hochberg Y. Controlling the False Discovery Rate: A Practical and Powerful Approach to Multiple Testing. Journal of the Royal Statistical Society: Series B (Methodological). 1995;57(1):289–300.

7. Karp NA, Mason J, Beaudet AL, Benjamini Y, Bower L, Braun RE, et al. Prevalence of sexual dimorphism in mammalian phenotypic traits. Nat Commun. 2017 Jun 26;8(1):15475.

8. Altman DG, Bland JM. Treatment allocation by minimisation. BMJ. 2005 Apr 9;330(7495):843.

9. Lenth, R. V. Java Applets for Power and Sample Size [Computer software] [Internet]. 2006. Available from: https://homepage.stat.uiowa.edu/~rlenth/Power/oldversion.html Accessed 04/2023.
