# Supplementary material for: Statistical simulations show that scientists need not increase overall sample size by default when including both sexes in in vivo studies
Source: PLoS Biol. 2023 Jun 8;21(6):e3002129. doi: 10.1371/journal.pbio.3002129 (PMC10284409; doi:10.1371/journal.pbio.3002129)
Supplement: Attachment — Submitted filename: phillips_responses_reviewers.docx [file pbio.3002129.s002.docx]

Dear Roland,

Thank you for your letter regarding our article “Statistical simulations show that scientists need not increase overall sample size by default when including both sexes in in vivo studies”.

We were pleased with the positive reactions of the reviewers and have found their feedback very helpful to refine the article further. We provide a revised version that we believe addresses the reviewers’ comments as detailed below. We look forward to hearing from you regarding this reworked manuscript.

Best wishes,

Natasha Karp

**EDITOR’S COMMENTS:**

**Please attend to the requests from the reviewers.**

**b) Regarding reviewer #1's comment "Perhaps another Article Type is more appropriate?," the answer is "yes" - I hadn't noticed that this was submitted as a regular Research Article; I have now switched it to a Meta-Research Article, which we feel should address this reviewer's valid concern - the advance here is in meta-research, not statistics.**

We agree with the editor and reviewers that the topic of the paper is focussed on meta-research, and that it would be more appropriate as a Meta-Research Article.  **c) Please change the Title to "Statistical simulations show that scientists need not increase overall sample size by default when including both sexes in in vivo studies"**

We are thankful for the suggestion and have changed the title accordingly.  **d) Please address my Data Policy requests below; specifically, we need you to supply the numerical values underlying Figs 1BCD, 2BCD, 3BCDE, 4BCD, S1ABC, either as a supplementary data file or as a permanent DOI’d deposition. I note that you already have a Zenodo deposition, but access is currently restricted, so I can’t check compliance; please rectify this.**

Thank you. We have made the Zenodo repository publicly available.

**e) Please cite the location of the data clearly in all relevant main and supplementary Figure legends, e.g. “The data underlying this Figure can be found in S1 Data” or “The data underlying this Figure can be found in** [**https://doi.org/10.5281/zenodo.7629021**](https://protect-de.mimecast.com/s/BT8WC46Aq2ClE0noEUXN9Wp?domain=doi.org)**”**

Thank you for the helpful suggestion. We have added “The data underlying this Figure can be found in https://doi.org/10.5281/zenodo.7806724 to each of the figure legends.

**REVIEWERS' COMMENTS:**


Reviewer #1:

**Phillips et al. use simulations to show that including animals of both sexes and using an appropriate statistical analysis does not reduce the power to detect treatment effects, compared with using animals of one sex. The authors identify two misconceptions held by many scientists and three errors that are frequently observed in published papers. They then use simulations to demonstrate why the misconceptions are incorrect and provide better ways to analyse data.

This paper has been submitted as "Research Article" and I have been asked to comment on the "novelty and significance of the findings, its technical merit, the and experimental design". All the results are expected based on statistical theory and are well known to statisticians, and so are not novel or significant. However, demonstrating the results with simulations may be more convincing to many scientists. Having such a paper to reference when discussing these issues with colleagues and collaborators would be valuable. Perhaps another Article Type is more appropriate?

The statistical analyses are appropriate and the supplementary information provides a useful case study with real data. I cannot access the scripts and data on Zenodo as access is restricted.**

We are thankful to the reviewer for their positive evaluation of our paper and helpful suggestions. We agree that publication as a Meta-Research Article is more appropriate.

**I only have a few minor comments:

1) The font size is too small in all the figures and difficult to read. Adding + theme(text = element_text(size = 14)) to the plots should fix this.**

Thank you for noting this issue with the readability of the plots. We have updated the figures with the requested change to improve clarity.

**2) Line 210: It would be useful to add Gelman and Stern's paper to the references here (**[**https://www.tandfonline.com/doi/abs/10.1198/000313006X152649**](https://protect-de.mimecast.com/s/d31dCgpRGySGK0jkKiYtFHi?domain=tandfonline.com)**). I believe they were the first to draw attention to this problem.**

Thank you for highlighting this important reference. We have updated the text and reference list to include the Gelman and Stern paper as suggested.

**3) Line 419-421: I think this sentence is missing some words. Is the intended meaning: "There is no requirement to perform a power calculation to detect ..."?**

We thank the reviewer for highlighting the lack of clarity in this section. We have updated the text as follows “There is no requirement to prospectively power a study to detect a baseline difference…” and believe that this will clarify the intended meaning.  **4) Panel A in Figure 2 is referenced multiple times (lines 191, 193, 198, 200) but it's unclear where we should be looking as there are four graphs. Adding a more specific pointer would make it clearer (e.g. Fig 2A, top right).**

We are grateful to the reviewer for highlighting the lack of clarity around this figure, and have amended the flags to point to specific graphs accordingly.


Reviewer #2:

**This is another outstanding and immensely practical contribution from this group. The paper does an excellent job in identifying the main issues, myths and all-too-common procedural errors involved in SABV studies. They provide transparent and readily reproducible methodology and code. I especially loved the explanatory boxes which made the methods clear without interrupting the flow of the text. 

Ethical oversight assurances are provided, and most animal-based procedures reported in accordance with ARRIVE 2.0. However, there was no statement as to the eventual disposition of the mice, and how they were euthanased prior to collecting kidney tissue samples. Please provide description of these methods.**

We are very grateful to the reviewer for their positive evaluation of our manuscript. Thank you for the helpful suggestions. We have added additional information on the method of sacrifice in the methods section of the supplementary material.

**A few other minor comments:** 

**1. The worked example is a really good idea. It could be made more practical for the average reader by streamlining the text quite a bit. The text is a bit clunky and focuses a lot on the results and less on the actual 'pipeline'. The text could be reduced by about 1/3rd & more bullet points added so it is easier to follow. Could the authors also consider placing this more upfront & incorporate something in the title & intro that states that the authors provide step by step methods on how to implement these features in actual studies (e.g. "tutorial" or "primer" into the title?) Or maybe consider a second companion paper? I am a bit worried that researchers who really need to see this will be put off by the heavy emphasis on the simulations & lessen the value of the practical implementation tips.**

We are grateful to the reviewer for their feedback on the worked example. The reviewer raises a critical point that errors in the analysis of factorial data are very common and require addressing. The case study described in this manuscript underpin (characteristics of the real data including the mean, variance, and effect sizes) the simulations described in the main paper but aims to provide a “light-touch” example of how data could be analysed. Whilst we see the importance of training scientists in factorial design, it is beyond the scope of the current manuscript to provide an exhaustive resource. Furthermore, whilst scientists frequently commit errors in formal statistical analysis of factorial designs, there is an additional important “skill gap” in interpretation of correctly applied statistical output. The case study is therefore aimed at exploring both aspects.

We currently have another initiative testing a training intervention (which include materials presented in the manuscript) and aim to develop an online course. We have reviewed the material to ensure clarity and retained a focus both on statistical implementation and biological interpretation.

**2, Although the authors incorporated bias minimisation methods such as allocation concealment and randomisation in their own studies, there is almost no mention up front. These are essential components of experimental design.**

We thank the reviewer for highlighting this important aspect of robust experimental design. We have added the following statement to the introductory section of the case study:

“We subsequently performed an experimental study where animals of each sex were allocated using an experimenter-masked (blinded) minimisation randomisation procedure, based on body weight, to treatment groups.”

**3. There is the added effect of cage. Was cage included as a block or were cage effects assumed to be zero?**

Thank you for noting cage as a potential source of variability. We have added the following explanatory statement in the statistical methods:

“Cage was not included as a factor in the model. This position was taken after minimizing practical cage differences (e.g., cage positions in rack) and the random allocation of animals to cage.”

**4. The sample size justification statement is far too vague to be verifiable. A few sentences could be added to explain. Not saying that it is, but as it stands, it seems to reinforce the all-too-common practice of choosing 5 per "group" because that is the usual per-cage stocking density.**

We thank the reviewer for pointing out our omission. We have added the following explanatory text in the methods:

“Specifically, we estimated power in terms of standard deviation unit change using the two sample *t*-test calculator of the Russ Lenth power tool (9). In this calculation, we estimated power by approximating the main effect as a two-tailed two group comparison at the 0.05 significance threshold and found that a N of 10 per treatment group (5 males and 5 females) would give us 0.8145 power to detect a change equivalent to 1.35 standard deviation units.”

**5. Mention should be made of sample size estimation in general for factorials. Powering factorials is different from the usual approach taken for two-group or single-factor completely randomised designs. As you know, main effects are estimated by comparing the means of 'sets of conditions' or a combination run, not by directly comparing the means of individual "groups". This needs to be mentioned upfront as well. Much of the resistance to adopting factorial designs is when researchers try to power them from G*Power which suggests far larger samples sizes are required. Sample sizes will also be too small for pairwise comparison of "groups". it is far more fruitful and less confusing to clarify that with factorials what is replicated are experimental runs (factor combinations) & that the researcher needs to think about total sample size, not group size. This is mentioned (sort of) in the text but it needs to be stated much more clearly. mention might be made of the Lakens and Caldwell paper (2021) on sample size for factorials. DOI:10.1177/2515245920951503**

We are grateful to the reviewer for pointing out the important topic of sample size calculation for factorial designs. We have clarified our advice in the concluding remarks and cited the Lakens and Caldwell paper as an alternative strategy for calculating power for factorial designs as follows:

“This strategy implies that power calculations for the treatment effect can be simplified to a 2-group comparison to estimate the total N needed for a treatment, which is then shared between the 2 sexes. Alternatively, power calculations for factorial designs can be calculated using other methods (e.g., the *Superpower* package (25))”

**6. If the factorial is conducted for screening purposes, emphasis should be on how "relative promise" of the sex x trt interactions are evaluated e.g in terms of precision of the estimate. not statistical significance. mention of practical effect size could be added up front.**

Whilst we agree with the reviewer that in situations in which the power for interaction terms is low, it is important to consider the biological significance of the estimate. However, this manuscript is centred around the premise of including both sexes to meet the sex inclusive philosophy and estimate a generalisable effect.  This does mean the power to detect interaction effects will be low.  We have added to the limitations section the following sentence:

“Critically, the scope of this manuscript is limited to studies where the intention is to estimate a generalisable treatment effect, rather than exploring the dependence of a treatment effect on sex.”


**Reviewer #3:

Summary: In this manuscript, Philips and Colleagues conduct statistical simulations to address common misconceptions surrounding the use of both sexes in in vivo studies. It is commonly thought that in order to design an experiment which is statistically powered to analyze data by sex, the sample size (n) must double. To address this, they conduct power analyses which mirror a variety of experimental conditions with varied effects of treatment and sex. They provide clear explanations surrounding the power analyses and the results. Their simulations highlight the fact that in most scenarios, when sexes are split across treatment groups, the sample size is enough to sufficiently power the study. 

Added Value of the Study: Several other articles have discussed how to design and analyze experiments involving both sexes. Most articles are written as reviews and provide overviews and/or tutorials of statistical methods. This manuscript expands upon that work by including the aforementioned power analyses and provides a case study which can be used for training purposes. It also reiterates the importance of factorial vs. pooled analyses, demonstrating that their incorrect use could lead to misleading results that ultimately detract from our understanding of how sex informs health and disease. 

Overall, I find this article to be well written and easily accessible to a broad audience. However, I acknowledge that I do not have expertise in statistical modeling and hope that other reviewers can comment on strengths and/or weaknesses that pertain to the methods used here.**

We are very grateful to the reviewer for their positive evaluation of our manuscript and are pleased that they feel it will add value in the literature.
